# Letter from the Editor in Chief

**DOI:** 10.19102/icrm.2022.13122

**Published:** 2022-12-15

**Authors:** Moussa Mansour


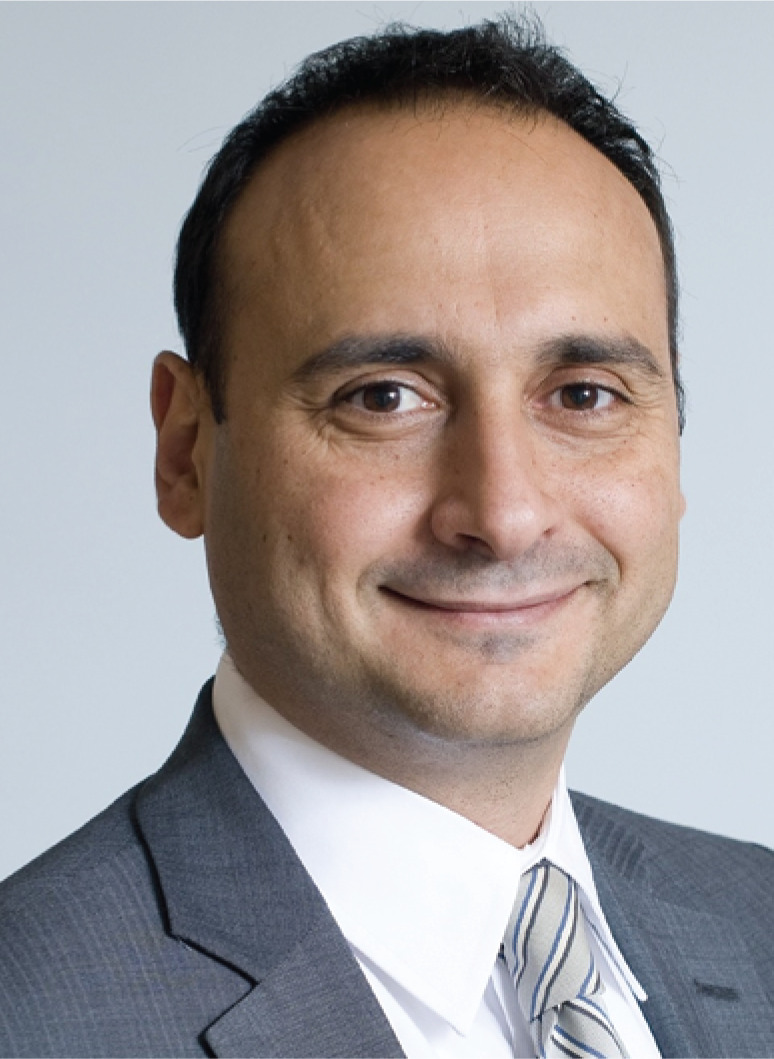


Dear readers,

During the recent annual scientific meeting of the American Heart Association in November, important late-breaking clinical trials, including the Impact of “First-Line” Rhythm Therapy on AF Progression (PROGRESSIVE-AF) study, were presented. In this study,^1^ 303 patients with untreated paroxysmal atrial fibrillation (AF) were randomly assigned to undergo initial rhythm-control therapy with cryoballoon ablation or receive anti-arrhythmic drug (AAD) therapy; during the 3-year follow-up period, 1.9% in the cryo-ablation group and 7.4% in the AAD group had ≥1 episodes of persistent AF (hazard ratio, 0.25; 95% confidence interval [CI], 0.09–0.70).

The PROGESSSIVE-AF study corroborated the results of the Atrial Fibrillation Progression Trial (ATTEST) study,^2^ which was stopped early due to slow enrollment but succeeded in enrolling 255 patients with paroxysmal AF who were randomized to either radiofrequency catheter ablation or AAD therapy. During 3 years of follow-up, 2.4% of the patients in the ablation group developed persistent AF (95% CI, 0.6–9.4) compared to 17.5% in the AAD group (95% CI, 10.7–27.9; 1-sided *P* = .0009). There were some small differences in the design of these studies, which might explain the slightly relative difference between the ablation and AAD groups. While ATTEST enrolled patients who had failed to respond with AAD therapy, patients in PROGRESSIVE-AF were previously untreated. Another difference is the mode of follow-up: all patients in PROGRESSIVE-AF had implantable loop recorders, while ATTEST relied on trans-telephonic monitoring. However, the result of the primary endpoint was similar in both studies, demonstrating a previously unknown benefit of early intervention in AF: a reduction in the rate of progression from paroxysmal to persistent AF.

The progression from paroxysmal to persistent AF likely involves electrical and structural remodeling of left atrial areas outside the pulmonary veins, which explains the lower success rate observed with pulmonary vein isolation (PVI) in paroxysmal versus persistent AF. Previous studies reported that early PVI for paroxysmal AF lowers the rate of AF recurrence during 1 year of follow-up compared to AAD treatment. ATTEST and PROGRESSIVE-AF further expanded this benefit and showed that early intervention with PVI also reduces the rate of progression to persistent AF. Having fewer patients with persistent AF may result in less-complicated ablation procedures, which will translate into higher long-term success rates and fewer complications.

Best wishes for a happy and healthy holiday season.


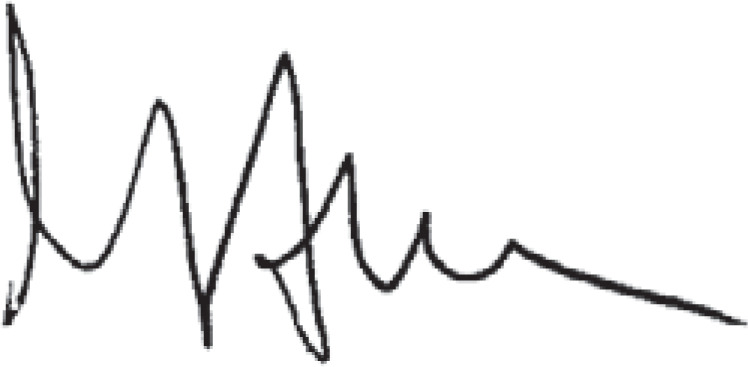


Sincerely,

Moussa Mansour, md, fhrs, facc

Editor in Chief

*The Journal of Innovations in Cardiac Rhythm Management*

MMansour@InnovationsInCRM.com

Director, Atrial Fibrillation Program

Jeremy Ruskin and Dan Starks Endowed Chair in Cardiology

Massachusetts General Hospital

Boston, MA 02114

## References

[1] Andrade JG, Deyell MW, Macle L Progression of atrial fibrillation after cryoablation or drug therapy [published online ahead of print November 7, 2022]. N Engl J Med.

[2] Kuck KH, Lebedev DS, Mikhaylov EN (2021). Catheter ablation or medical therapy to delay progression of atrial fibrillation: the randomized controlled atrial fibrillation progression trial (ATTEST). Europace.

